# Comparative Effectiveness of Continuous Intra-Operative Suprascapular Nerve Block (CI-SSNB) with and Without Intravenous Patient-Controlled Analgesia (IV-PCA) on Acute Post-Arthroscopy Pain: A Retrospective Cohort Study

**DOI:** 10.3390/jcm14165809

**Published:** 2025-08-16

**Authors:** Sung-yup Hong, Dong-woo Lee, Ji-hun Kim, Yoon-suk Hyun

**Affiliations:** Department of Orthopedic Surgery, Kangdong Sacred Heart Hospital, Hallym University, Seoul 05355, Republic of Korea; 110226@kdh.or.kr (S.-y.H.); ldw93@kdh.or.kr (D.-w.L.); smithwill4852@kdh.or.kr (J.-h.K.)

**Keywords:** intravenous patient-controlled analgesia (IV-PCA), continuous intra-operative suprascapular nerve block (CI-SSNB), arthroscopic rotator cuff repair (ARCR), interscalene block (ISB), visual analog scale (VAS), post-operative nausea and vomiting (PONV)

## Abstract

**Background/Objectives:** Intravenous patient-controlled analgesia (IV-PCA) is commonly used for pain control following arthroscopic rotator cuff repair (ARCR), but its use is limited by adverse effects such as nausea and vomiting. The suprascapular nerve block (SSNB) has emerged as an effective regional analgesic alternative. This retrospective cohort study aimed to compare the analgesic efficacy and safety of continuous intra-operative suprascapular nerve block (CI-SSNB) alone versus CI-SSNB combined with fentanyl-based IV-PCA (CI-SSNB + IV-PCA). **Methods:** A total of 40 patients undergoing ARCR under general anesthesia with a single-shot interscalene block (ISB) were allocated to either CI-SSNB alone (*n* = 20) or CI-SSNB + IV-PCA (*n* = 20). Pain scores were assessed using a 0–10 visual analog scale from 0 to 72 h postoperatively at predetermined intervals, along with opioid consumption and adverse events. **Results:** At post-operative day 0 (POD 0, 10 p.m.), mean pain scores were 5.75 ± 2.59 in the CI-SSNB + IV-PCA group vs. 3.95 ± 3.00 in the CI-SSNB group (*p* = 0.050). The total number of rescue pethidine doses up to post-operative day 3 was 1.80 ± 2.02 vs. 0.95 ± 1.10, respectively (*p* = 0.108). However, adverse effects such as nausea and vomiting occurred only in the CI-SSNB + IV-PCA group. **Conclusions:** CI-SSNB provides comparable analgesia to CI-SSNB + IV-PCA, while avoiding IV-PCA-related side effects, suggesting that IV-PCA may not be necessary when CI-SSNB is employed for post-operative analgesia following ARCR.

## 1. Introduction

Adequate post-operative pain control following arthroscopic rotator cuff repair (ARCR) of the shoulder is essential for patient rehabilitation, functional recovery, and early restoration of range of motion [1]. As a result, the interscalene brachial plexus block (ISB) has become the conventional modality for post-operative pain control [2].

Single-shot ISB is widely utilized for peri-operative analgesia in shoulder surgery; however, it is frequently associated with adverse events such as dyspnea, hoarseness, inadequate anesthetic effect, and dizziness [3]. Additionally, its analgesic efficacy typically diminishes approximately 24 h post-procedure [4,5]. To manage ongoing pain after ISB subsides, intravenous patient-controlled analgesia (IV-PCA) is commonly administered. Nonetheless, IV-PCA frequently induces significant adverse effects—particularly post-operative nausea and vomiting [6,7]—leading to patient discomfort, premature discontinuation, inadequate pain relief, and increased healthcare costs.

To extend analgesic coverage beyond the limited duration of ISB without encountering the adverse effects associated with IV-PCA, clinicians have explored alternative strategies such as continuous intra-operative suprascapular nerve block (CI-SSNB) [8,9]. Previous comparative studies indicate that CI-SSNB alone might be insufficient, with many patients still requiring supplemental opioid rescue medications; however, combining CI-SSNB with ISB seems to improve early post-operative pain control [10]. Recent studies also suggest that CI-SSNB can mitigate the “rebound” pain observed after the analgesic effects of ISB dissipate [1].

In our institution, all patients routinely receive a single-shot ISB as part of the anesthetic protocol for ARCR, in addition to CI-SSNB. Therefore, the present study specifically compared patients undergoing ARCR who received ISB + CI-SSNB versus ISB + CI-SSNB with the addition of IV-PCA. We aimed to evaluate whether CI-SSNB combined with ISB can provide sufficient analgesia without the need for IV-PCA, thereby minimizing opioid-related side effects, enhancing patient comfort, and improving cost-effectiveness.

## 2. Materials and Methods

### 2.1. Study Design and Population

This retrospective cohort study was conducted in accordance with the Declaration of Helsinki and was approved by the Institutional Review Board of Kangdong Sacred Heart Hospital (IRB No. 2025-06-003, date: 12 June 2025). The requirement for informed consent was waived given the retrospective nature of the investigation. Adult patients who underwent elective arthroscopic rotator cuff repair (ARCR) between December 2024 and May 2025 were reviewed. Patients who received continuous intra-operative suprascapular nerve block (CI-SSNB) in combination with single-shot interscalene brachial plexus block (ISB) for post-operative analgesia were eligible for inclusion. Based on the use of adjunctive intravenous patient-controlled analgesia (IV-PCA), patients were categorized into two groups: ISB + CI-SSNB group and ISB + CI-SSNB + IV-PCA group. Group allocation was determined based on patient preference following pre-operative counseling, which included explanation of the potential benefits, risks, and additional costs associated with IV-PCA. As both regimens were considered acceptable standards of care in our institution, patients who declined IV-PCA due to personal preference or financial considerations were included in the CI-SSNB group. Aside from the use of IV-PCA, all patients received a standardized anesthetic regimen preoperatively and intraoperatively to minimize variability between groups.

**Figure 1:**
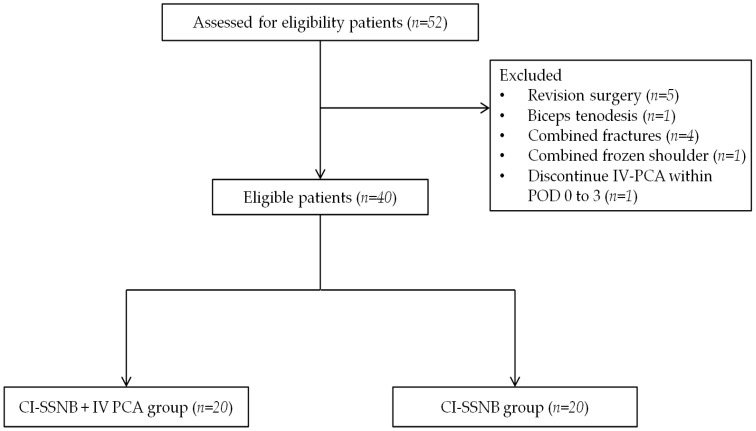
Patient flowchart. CI-SSNB, continuous intra-operative suprascapular nerve block; IV-PCA, intravenous patient-controlled analgesia; POD, post-operative day.

Exclusion criteria were as follows: (1) patients who underwent concomitant procedures involving the affected shoulder, such as revision surgery, biceps tenodesis, fracture fixation, or capsular release for frozen shoulder; and (2) patients who permanently discontinued IV-PCA due to systemic adverse effects—such as nausea or vomiting—within post-operative days 0 to 2. Patients who experienced such adverse effects but resumed IV-PCA after anti-emetic treatment, or continued IV-PCAs with bolus-only suspension, were not excluded (Figure 1).

### 2.2. Operative Technique of SSNB

All procedures were performed by the single orthopedic surgeon with subspecialty training in shoulder surgery, with patients placed in the beach-chair position under general anesthesia. Prior to induction, a single-shot, ultrasound-guided interscalene brachial plexus block (ISB) was performed by an anesthesiologist using a total of 18 mL of anesthetic solution, consisting of 6 mL of 0.75% ropivacaine (7.5 mg/mL), 6 mL of 2% lidocaine (20 mg/mL), and 6 mL of normal saline.

**Figure 2:**
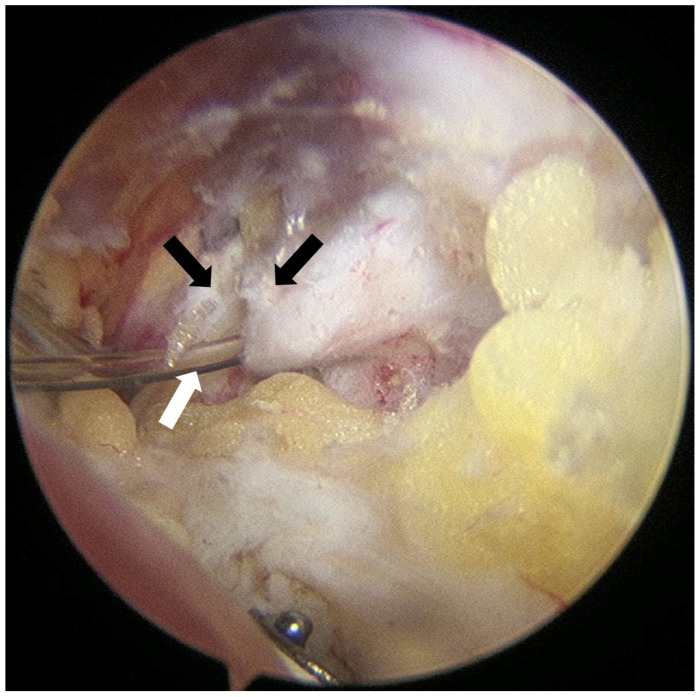
Arthroscopic image of catheter insertion procedure after transverse ligament release. The superior transverse scapular ligament (black arrows) was carefully released with an arthroscopic scissor, followed by the insertion of an epidural catheter (white arrow). The suprascapular nerve courses beneath the ligament.

After subacromial bursectomy, a Neviaser portal was established. With the arthroscope placed in the posterior portal to visualize the suprascapular notch, the superior transverse scapular ligament (STSL) was cut with arthroscopic scissors. Once a guide needle reached the posterior surface of the STSL, a 19-gauge FX Springwound Epidural Anesthesia Catheter (Perifix^®^, B. Braun, Penang, Malaysia) was inserted via needle through the Neviaser portal and advanced approximately 1 cm beyond the posterior border of the STSL to prevent catheter displacement (Figure 2). The catheter tip was positioned adjacent to the suprascapular nerve at the base of the suprascapular notch, and fixation was secured using 3-0 nylon tagging suture. Rotator cuff repair was subsequently performed using a standard suture-bridge technique.

### 2.3. Post-Operative Analgesic Protocol

**Figure 3:**
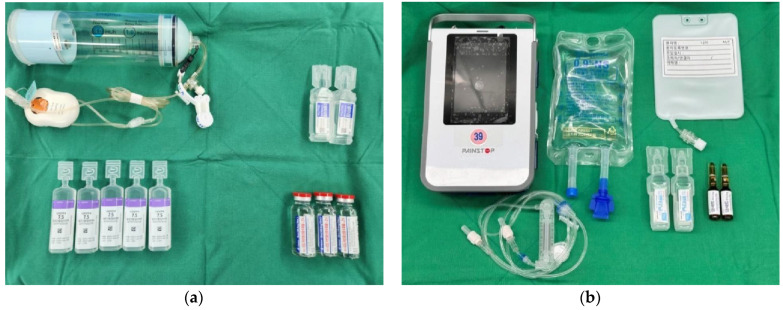
Post-operative analgesic preparation sets (**a**) Components used for continuous intra-operative suprascapular nerve block (CI-SSNB), including an elastomeric pump, local anesthetics (2% lidocaine, 0.75% ropivacaine), and normal saline. (**b**) Materials required for intravenous patient-controlled analgesia (IV-PCA), including the PCA device, fentanyl-based solution, ramosetron hydrochloride, IV lines, and normal saline.

Immediately following arthroscopic surgery, a CI-SSNB infusion was initiated using an elastomeric pump. The infusion solution consisted of 60 mL of 2% lidocaine (20 mg/mL), 100 mL of 0.75% ropivacaine (7.5 mg/mL), and 40 mL of normal saline, yielding a total volume of 200 mL. This mixture was continuously infused at a rate of approximately 4.17 mL/h (Figure 3a). Patients were instructed to self-administer a bolus dose by pressing a button on the pump in response to pain. The infusion system was refilled as needed and maintained until the morning of post-operative day (POD) 3 to maintain consistent pain relief.

In the CI-SSNB + IV-PCA group, patients received a fentanyl-based intravenous patient-controlled analgesia (IV-PCA) regimen consisting of a continuous basal infusion at 1 µg·kg^−1^·h^−1^, a 15 µg bolus dose, and a 15 min lockout interval (Figure 3b). The device was maintained until the morning of POD 3, and patients were allowed to self-administer bolus doses for breakthrough pain. Intravenous pethidine (25 mg) was provided as rescue analgesia when the visual analog scale (VAS) score was ≥4 upon patient request.

In cases where IV-PCA–related side effects such as nausea or vomiting occurred, bolus administration was initially suspended while maintaining the basal infusion. If symptoms persisted, continuous infusion was also temporarily halted. Anti-emetic medications were administered as needed, and the decision to resume or discontinue IV-PCA was made according to the patient’s clinical condition and response to treatment.

### 2.4. Outcome Measures

The primary outcome was rest pain VAS at six predefined intervals: post-anesthesia care unit (PACU), 10 p.m. on the day of surgery, and 9 a.m. on post-operative day (POD) 1 to POD 3. Secondary outcomes included cumulative rescue opioid consumption (converted to IV morphine equivalents), IV-PCA device events (pump alarm), and post-operative nausea and vomiting (PONV).

### 2.5. Statistical Analysis

Continuous data are reported as mean ± standard deviation (SD) and compared using the Mann–Whitney U-test; categorical data were analyzed using the χ^2^ test or Fisher’s exact test, as appropriate. Two-sided *p*-value < 0.05 was considered statistically significant. All statistical analyses were performed using Python 3.10 (SciPy 1.11) and IBM SPSS Statistics version 28.0 (IBM Corp., Armonk, NY, USA).

## 3. Results

### 3.1. Baseline Characteristics

A total of 40 patients met the inclusion criteria and were allocated evenly into the CI-SSNB + IV-PCA group (*n* = 20) and the CI-SSNB-only group (*n* = 20). Demographic and surgical characteristics were generally comparable between the two groups (Table 1). The mean age was 63.2 ± 8.9 years in the CI-SSNB + IV-PCA group and 64.2 ± 6.3 years in the CI-SSNB group (*p* = 0.685). The sex distribution was similar, with 65.0% versus 70.0% male patients, respectively (*p* = 1.000). Although the mean body mass index was slightly lower in the CI-SSNB + IV-PCA group (24.2 ± 6.5 kg/m^2^) compared to the CI-SSNB group (27.3 ± 2.8 kg/m^2^), the difference did not reach statistical significance (*p* = 0.060). Regarding fatty degeneration of the rotator cuff muscles, as classified by Goutallier grade, the majority of patients in both groups were classified as grade 1 or 2, with no significant between-group differences (*p* = 0.798). The mean cuff tear size was 1.72 ± 0.97 cm in the CI-SSNB + IV-PCA group and 1.43 ± 0.97 cm in the CI-SSNB group (*p* = 0.354). ASA physical status classification was also comparable between groups (*p* = 0.749), with most patients graded as ASA 2. Mean operative time did not differ significantly, being 129.4 ± 30.5 min versus 125.1 ± 45.4 min, respectively (*p* = 0.730). Overall, no statistically significant differences were observed between groups, confirming adequate baseline comparability.

### 3.2. Pain Scores

**Figure 4:**
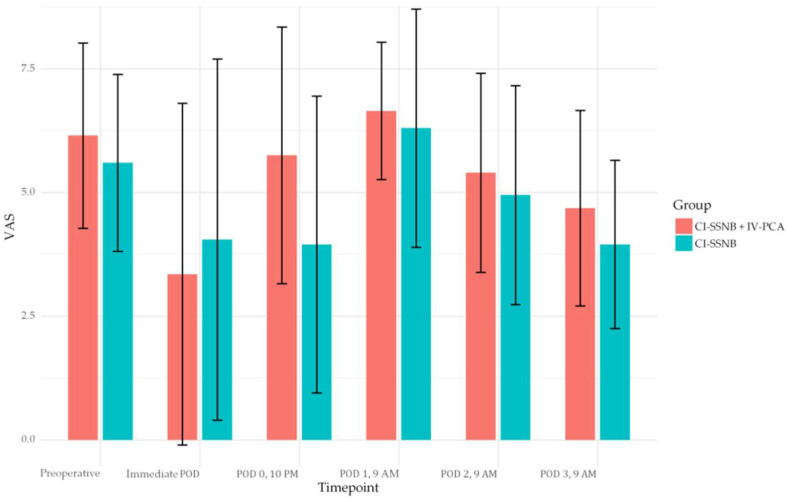
Bar-plot of Visual analog scale, mean VAS with standard deviations were reported at each post-operative timepoint for both CI-SSNB + IV PCA and CI-SSNB groups. VAS, visual analog scale; CI-SSNB, continuous intra-operative suprascapular nerve block; IV-PCA, intravenous patient-controlled analgesia.

Mean VAS values were comparable between the two groups across all assessed post-operative intervals. Preoperatively, the IV-PCA + CI-SSNB group reported 6.15 ± 1.87 compared with 5.60 ± 1.79 in the CI-SSNB group (*p* = 0.348). Immediately after surgery, mean VAS values were 3.35 ± 3.45 and 4.05 ± 3.65, respectively (*p* = 0.537). At POD 0 (10 p.m.), mean VAS was 5.75 ± 2.59 in the IV-PCA + CI-SSNB group versus 3.95 ± 3.00 in the CI-SSNB group (*p* = 0.050). At POD 1 (9 a.m.), the scores were 6.65 ± 1.39 and 6.30 ± 2.41, respectively (*p* = 0.577). No statistically significant differences were observed at any other interval. Post-hoc power analysis demonstrated a maximum power of 52.8% at POD 0 (10 p.m.), with lower values at other time points. Detailed results are provided in Table 2 and illustrated in Figure 4.

### 3.3. Opioid Consumption and Adverse Events

Cumulative rescue opioid consumption did not differ significantly between the CI-SSNB + IV-PCA and CI-SSNB-only groups (1.80 ± 2.02 vs. 0.95 ± 1.10 doses, *p* = 0.108). As shown in Table 3, 9 patients (45.0%) in each group did not require any rescue opioid up to POD 3. Among those who required additional analgesia, the distribution of first pethidine use was not different between CI-SSNB + IV-PCA group and CI-SSNB group (*p* = 1.000), with most patients receiving their first dose on POD 0 or POD 1. Regarding total pethidine doses, a borderline difference was observed (*p* = 0.052), with 4 patients (20.0%) in the CI-SSNB + IV-PCA group receiving 5 doses.

A total of 3 PONV episodes and 4 pump alarms were recorded exclusively in the CI-SSNB + IV-PCA group. The pump alarms were attributed to either transient occlusion of the infusion line or minor technical malfunctions of the IV-PCA device. All events were promptly resolved without necessitating discontinuation of IV-PCA. Specifically, 2 cases of nausea were observed on post-operative day (POD) 1 and 1 case of vomiting occurred on POD 2. All symptoms were mild to moderate in severity and were effectively managed with intravenous antiemetics, after which the patients were able to continue IV-PCA without recurrence. No block-related complications were observed in either group (Table 4).

## 4. Discussion

This study investigated whether the addition of intravenous patient-controlled analgesia (IV-PCA) to continuous intra-operative suprascapular nerve block (CI-SSNB) could enhance post-operative pain control after arthroscopic rotator cuff repair (ARCR). Our findings demonstrated that combining IV-PCA with CI-SSNB did not result in superior analgesia compared to CI-SSNB alone. Pain scores between the two groups were statistically comparable at all measured time points, and cumulative opioid rescue requirements did not differ significantly (1.80 ± 2.02 vs. 0.95 ± 1.10 doses, *p* = 0.108). As shown in Table 3, nearly half of the patients in each group did not require any rescue opioid. Among those who required additional analgesia, the distribution of first pethidine use wasn’t different between groups (*p* = 1.000), and the total number of rescue doses showed a borderline difference (*p* = 0.052), with several patients in the CI-SSNB + IV-PCA group requiring up to five doses.

These results are noteworthy given that IV-PCA has traditionally been employed to maintain consistent analgesia following single-shot regional blocks such as interscalene or suprascapular nerve blocks [11]. However, despite this theoretical advantage, patients in the CI-SSNB + IV-PCA group did not experience better pain relief. Instead, the use of IV-PCA was associated with a greater incidence of systemic side effects, including nausea, vomiting, and urinary retention—adverse events that were entirely absent in the CI-SSNB group. Such side effects, although mostly mild to moderate, may contribute to increased discomfort and reduced tolerance of post-operative care, even in the absence of clear analgesic benefit [12]. Notably, two patients in the IV-PCA group required temporary suspension of continuous infusion because of intolerance, underscoring the clinical relevance of these adverse effects.

In particular, the borderline significance observed at the immediate post-operative interval (*p* = 0.050) may have been influenced not only by the residual analgesic effect of the single-shot interscalene block (ISB), which typically provides pain relief for 12–24 h, but also by several additional factors. Some patients received rescue analgesia in the post-anesthesia care unit, which may have lowered reported pain scores. Moreover, psychological reassurance from having an IV-PCA device might have temporarily changed patients’ pain perception [13]. Finally, although the anesthetic solution and dosage were standardized, the ISB was performed by one of three anesthesiologists, and subtle inter-operator differences may have affected block efficacy. Together, these factors could explain the near-significant finding at this early time point.

Our findings align with previous randomized controlled trials that have highlighted the analgesic benefits of suprascapular nerve block–based strategies. Choi et al. (2020) demonstrated that continuous suprascapular nerve block achieved significantly lower VAS pain scores at 6–12 h postoperatively compared to single-shot interscalene block in ARCR patients [14]. Similarly, Kim et al. (2021) reported that an indwelling SSNB catheter resulted in lower immediate post-operative pain and reduced opioid requirements versus single-shot ISB, with fewer rebound pain episodes [1]. Unlike these trials, which primarily investigated post-operative catheter placement or single-shot techniques, our study uniquely evaluated intra-operative placement of a continuous suprascapular nerve block with or without IV-PCA, allowing infusion to commence during surgery. By demonstrating that CI-SSNB alone provides sufficient analgesia while avoiding IV-PCA–related side effects, our results add novel clinical insight into the optimization of multimodal pain management protocols for ARCR.

Interestingly, although the differences were not statistically significant, pain scores in the CI-SSNB group were numerically lower than those in the combination group at several post-operative time points. This paradoxical trend may be explained by several factors. First, the waning effect of the interscalene block, which generally lasts 12–24 h, could have resulted in rebound pain, potentially accentuated in the IV-PCA group where systemic opioids were not always sufficient to compensate for the loss of regional coverage. Second, opioid-related side effects such as nausea or dizziness may have negatively influenced patients’ subjective perception of pain despite analgesic administration. Third, psychological expectations regarding IV-PCA efficacy could have led to increased requests for rescue opioids when perceived relief was insufficient. Supporting this, our subgroup analysis excluding patients who did not require rescue opioids demonstrated a statistically significant higher total consumption of pethidine in the IV-PCA + CI-SSNB group (*p* = 0.052). These findings highlight that IV-PCA, rather than enhancing analgesia, may paradoxically increase opioid use in patients who are already receiving effective regional anesthesia.

Given these findings, our study suggests that while CI-SSNB alone may offer a comparable level of analgesia to the combined use of IV-PCA, it may not be sufficient to achieve optimal post-operative pain control in patients undergoing ARCR. Pain scores remained relatively elevated in both groups during the early post-operative period, indicating a need for further refinement of current analgesic strategies. Eliminating routine IV-PCA use may help reduce opioid-related adverse effects and simplify post-operative care, particularly in elderly or opioid-sensitive populations. Nevertheless, pain scores remained relatively elevated in both groups during the early post-operative period, suggesting that CI-SSNB, while effective, may not completely address all post-operative discomfort. Additional strategies such as multimodal non-opioid adjuncts or optimized catheter placement could be explored in future studies to further enhance pain control. Further research is warranted to establish more effective and tolerable analgesic strategies for ARCR.

This study has several limitations. First, its retrospective design introduces potential selection bias and limits the ability to infer causality. Second, the sample size was relatively small, and post-hoc power analysis indicated limited statistical power (maximum 52.8% for the primary outcome), reducing the ability to detect subtle but clinically meaningful differences. Third, although the CI-SSNB technique was standardized, inter-operator variability among anesthesiologists performing the interscalene block could have influenced block efficacy. Fourth, we did not evaluate functional outcomes such as early range of motion or sleep quality, as post-operative immobilization in a shoulder abduction brace for six weeks is standard after ARCR [15], precluding meaningful functional assessment during the immediate post-operative period. Nevertheless, sleep quality is an important patient-centered outcome that can be significantly influenced by post-operative pain, discomfort from immobilization, and opioid-related side effects such as nausea or dizziness [16,17,18]. The absence of a standardized sleep quality assessment in this study represents an additional limitation. Future prospective studies should incorporate validated patient-reported outcome measures for sleep quality and related functional recovery to provide a more comprehensive assessment of the clinical impact of different analgesic strategies. Finally, long-term outcomes were not assessed, limiting conclusions regarding durability of analgesic efficacy.

Despite these limitations, the present study adds to the growing body of evidence that continuous suprascapular nerve block, when combined with a single-shot interscalene block, can provide effective analgesia for ARCR without the need for routine IV-PCA. Our findings support the selective rather than routine use of IV-PCA, reserving it for patients at higher risk of inadequate pain control or significant rebound pain. Future large-scale prospective studies are warranted to validate these findings and to explore the integration of CI-SSNB into multimodal analgesic protocols tailored to patient risk profiles.

## 5. Conclusions

In this retrospective study, we found that continuous intra-operative suprascapular nerve block (CI-SSNB) provided post-operative analgesia comparable to that achieved with the combined use of CI-SSNB and intravenous patient-controlled analgesia (IV-PCA) in patients undergoing arthroscopic rotator cuff repair (ARCR). Although both strategies were effective in reducing post-operative pain, the addition of IV-PCA did not result in superior analgesic outcomes and was associated with a higher incidence of systemic adverse effects, including nausea, vomiting, and urinary retention—events that were not observed in the CI-SSNB group. While nearly half of patients in each group required no rescue opioids, subgroup analysis revealed that among those who did, patients in the IV-PCA group tended to consume more pethidine, with some requiring up to 5 doses. This paradoxical finding may reflect the influence of opioid-related side effects, rebound pain following the resolution of interscalene block, or variability in IV-PCA utilization. These findings suggest that the routine implementation of IV-PCA may be unnecessary when effective regional analgesia with CI-SSNB is provided.

Despite these observations, absolute pain scores in both groups remained higher than anticipated during the early post-operative period, suggesting that neither regimen provided fully satisfactory analgesia. These results underscore the need for further refinement of post-operative pain management strategies, including optimization of regional techniques, adjustment of infusion parameters, and the selective incorporation of multimodal non-opioid adjuncts.

Future large-scale randomized controlled trials are warranted to confirm these results, to evaluate long-term functional and patient-reported outcomes, and to further establish the role of CI-SSNB-focused analgesic protocols in arthroscopic shoulder surgery.

## Figures and Tables

**Table 1:** Patients Demographics (*n* = 40).

Variable		CI-SSNB + IV-PCA	CI-SSNB	*p*-Value
*n*		20	20	
Age		63.20 ± 8.93	64.20 ± 6.32	0.685
Sex (%)	Male	13 (65.0)	14 (70.0)	1.000
	Female	7 (35.0)	6 (30.0)	
BMI (kg/m^2^)		24.22 ± 6.49	27.33 ± 2.78	0.060
Goutallier Classification (%)	1	13 (65.0)	10 (50.0)	0.798
	2	5 (25.0)	7 (35.0)	
	3	2 (10.0)	2 (10.0)	
	4	0 (0.0)	1 (5.0)	
Cuff Tear Size (cm)		1.72 ± 0.97	1.43 ± 0.97	0.354
ASA (%)	1	2 (10.0)	4 (20.0)	0.749
	2	13 (65.0)	11 (55.0)	
	3	5 (25.0)	5 (25.0)	
Operation Time (min)		129.35 ± 30.52	125.10 ± 45.37	0.730

IV-PCA, intravenous patient-controlled analgesia; CI-SSNB, continuous intra-operative suprascapular nerve block; *n*, number; BMI, body mass index; ASA, American Society of Anesthesiologists.

**Table 2:** Mean VAS score.

Variable	CI-SSNB + IV-PCA	CI-SSNB	*p*-Value	Power
*n*	20	20		%
Preoperative	6.15 ± 1.87	5.60 ± 1.79	0.348	15.81
Immediate POD	3.35 ± 3.45	4.05 ± 3.65	0.537	9.55
POD 0, 10 p.m.	5.75 ± 2.59	3.95 ± 3.00	0.050	52.8
POD 1, 9 a.m.	6.65 ± 1.39	6.30 ± 2.41	0.577	8.71
POD 2, 9 a.m.	5.40 ± 2.01	4.95 ± 2.21	0.505	10.33
POD 3, 9 a.m.	4.68 ± 1.97	3.95 ± 1.70	0.222	24.27

VAS, visual analog scale; CI-SSNB, continuous intra-operative suprascapular nerve block; IV-PCA, intravenous patient-controlled analgesia; power, post-hoc power analysis; *n*, number; POD, post-operative day.

**Table 3:** Cumulative rescue opioid consumptions.

Variable		CI-SSNB + IV-PCA	CI-SSNB	*p*-Value
*n*		20	20	
First Pethidine Use Day (%)	No use	9 (45.0)	9 (45.0)	1.000
	POD 0	5 (25.0)	6 (30.0)	
	POD 1	5 (25.0)	4 (20.0)	
	POD 2	1 (5.0)	0 (0.0)	
	POD 3	0 (0.0)	1 (5.0)	
Total Pethidine Dose (%)	0 *	9 (45.0)	9 (45.0)	0.052
	1	1 (5.0)	6 (30.0)	
	2	4 (20.0)	2 (10.0)	
	3	1 (5.0)	3 (15.0)	
	4	1 (5.0)	0 (0.0)	
	5	4 (20.0)	0 (0.0)	

CI-SSNB, continuous intra-operative suprascapular nerve block; IV-PCA, intravenous patient-controlled analgesia; *n*, number; POD, post-operative day. *: The numbers 0 to 5 indicate the total number of intravenous pethidine rescue doses (25 mg each) administered to each patient during the first three post-operative days (POD 0 to 3). A value of 0 represents patients who did not require any pethidine during this period, while higher values correspond to the cumulative frequency of pethidine administration.

**Table 4:** IV-PCA side effects.

	Variable	CI-SSNB + IV-PCA	CI-SSNB	*p*-Value
IV-PCA Side Effect (%)	Headache	1 (5.0)	0 (0.0)	0.020
	Nausea	2 (10.0)	0 (0.0)	
	Vomiting	1 (5.0)	0 (0.0)	
	Urinary Retention	2 (10.0)	0 (0.0)	
	None	14 (70.0)	20 (100.0)	

IV-PCA, intravenous patient-controlled analgesia; CI-SSNB, continuous intra-operative suprascapular nerve block.

## Data Availability

The original contributions presented in this study are included in the article. Further inquiries can be directed to the corresponding author.

## References

[1] Kim J.Y., Kang M.W., Lee H.W., Noh K.C. (2021). Suprascapular Nerve Block Is an Effective Pain Control Method in Patients Undergoing Arthroscopic Rotator Cuff Repair: A Randomized Controlled Trial. Orthop. J. Sports Med..

[2] Warrender W.J., Syed U.A.M., Hammoud S., Emper W., Ciccotti M.G., Abboud J.A., Freedman K.B. (2017). Pain Management After Outpatient Shoulder Arthroscopy: A Systematic Review of Randomized Controlled Trials. Am. J. Sports Med..

[3] Tanijima M., Takechi K., Nakanishi K., Yorozuya T. (2019). Adverse events associated with continuous interscalene block administered using the catheter-over-needle method: A retrospective analysis. BMC Anesthesiol..

[4] White L., Reardon D., Davis K., Velli G., Bright M. (2022). Anterior suprascapular nerve block versus interscalene brachial plexus block for arthroscopic shoulder surgery: A systematic review and meta-analysis of randomized controlled trials. J. Anesth..

[5] Oh J.H., Rhee K.Y., Kim S.H., Lee P.B., Lee J.W., Lee S.J. (2009). Comparison of Analgesic Efficacy between Single Interscalene Block Combined with a Continuous Intra-bursal Infusion of Ropivacaine and Continuous Interscalene Block after Arthroscopic Rotator Cuff Repair. Clin. Orthop. Surg..

[6] Hah J.M., Bateman B.T., Ratliff J., Curtin C., Sun E. (2017). Chronic Opioid Use After Surgery: Implications for Perioperative Management in the Face of the Opioid Epidemic. Anesth. Analg..

[7] Dinges H.C., Otto S., Stay D.K., Bäumlein S., Waldmann S., Kranke P., Wulf H.F., Eberhart L.H. (2019). Side Effect Rates of Opioids in Equianalgesic Doses via Intravenous Patient-Controlled Analgesia: A Systematic Review and Network Meta-analysis. Anesth. Analg..

[8] Hussain N., Costache I., Kumar N., Essandoh M., Weaver T., Wong P., Tierney S., Rose P., McCartney C.J., Abdallah F.W. (2020). Is Supraclavicular Block as Good as Interscalene Block for Acute Pain Control Following Shoulder Surgery? A Systematic Review and Meta-analysis. Anesth. Analg..

[9] Sun C., Zhang X., Ji X., Yu P., Cai X., Yang H. (2021). Suprascapular nerve block and axillary nerve block versus interscalene nerve block for arthroscopic shoulder surgery: A meta-analysis of randomized controlled trials. Medicine.

[10] Liu Z., Li Y.B., Wang J.H., Wu G.H., Shi P.C. (2022). Efficacy and adverse effects of peripheral nerve blocks and local infiltration anesthesia after arthroscopic shoulder surgery: A Bayesian network meta-analysis. Front. Med..

[11] Cho N.S., Ha J.H., Rhee Y.G. (2007). Patient-Controlled Analgesia after Arthroscopic Rotator Cuff Repair:Subacromial Catheter Versus Intravenous Injection. Am. J. Sports Med..

[12] Scoggin III J.F., Mayfield G., Awaya D.J., Pi M., Prentiss J., Takahashi J. (2002). Subacromial and intra-articular morphine versus bupivacaine after shoulder arthroscopy. Arthrosc. J. Arthrosc. Relat. Surg..

[13] Patak L.S., Tait A.R., Mirafzali L., Morris M., Dasgupta S., Brummett C.M. (2013). Patient Perspectives of Patient-Controlled Analgesia (PCA) and Methods for Improving Pain Control and Patient Satisfaction. Reg. Anesth. Amp; Pain Med..

[14] Choi H., Roh K., Joo M., Hong S.H. (2020). Continuous suprascapular nerve block compared with single-shot interscalene brachial plexus block for pain control after arthroscopic rotator cuff repair. Clinics.

[15] Grubhofer F., Ernstbrunner L., Gerber C., Hochreiter B., Schwihla I., Wieser K., Bouaicha S. (2022). Effect of abduction brace wearing compliance on the results of arthroscopic rotator cuff repair. JBJS Open Access.

[16] Kim H., Lee J., Koh K.H. (2025). Effect of arthroscopic repair on sleep disturbances in rotator cuff tear patients: A prospective cohort study analyzing short-term postoperative pain correlations. J. Orthop. Surg..

[17] Zhang Q., Li Y., Li Y., Wang C., Yao Y., Li Q. (2024). Analysis of related factors influencing the sleep quality in patients with rotator cuff tear after arthroscopic surgery. Medicine.

[18] Perez A.R., Destiné H., Patel N.K., Campbell R.E., Muchintala R., Hall A.T., Pepe M.D., Tucker B.S., Tjoumakaris F.P. (2024). Effects of Melatonin on Sleep Quality and Patient-Reported Outcomes After Arthroscopic Rotator Cuff Surgery: A Prospective Randomized Controlled Trial. Am. J. Sports Med..

